# Reconstructed Global Invasion and Spatio-Temporal Distribution Pattern Dynamics of *Sorghum halepense* under Climate and Land-Use Change

**DOI:** 10.3390/plants12173128

**Published:** 2023-08-31

**Authors:** Ming Yang, Haoxiang Zhao, Xiaoqing Xian, Yuhan Qi, Qiao Li, Jianying Guo, Li Chen, Wanxue Liu

**Affiliations:** 1 State Key Laboratory for Biology of Plant Diseases and Insect Pests, Institute of Plant Protection, Chinese Academy of Agricultural Sciences, Beijing 100193, China; 2 School of Life Sciences, Hebei University, Baoding 071000, China

**Keywords:** climate change, *Sorghum halepense*, land-use change, potential global suitable habitats, invasive alien plants

## Abstract

*Sorghum halepense* competes with crops and grass species in cropland, grassland, and urban environments, increasing invasion risk. However, the invasive historical dynamics and distribution patterns of *S. halepense* associated with current and future climate change and land-use change (LUC) remain unknown. We first analyzed the invasive historical dynamics of *S. halepense* to explore its invasion status and expansion trends. We then used a species distribution model to examine how future climate change and LUC will facilitate the invasion of *S. halepense*. We reconstructed the countries that have historically been invaded by *S. halepense* based on databases with detailed records of countries and occurrences. We ran biomod2 based on climate data and land-use data at 5′ resolution, assessing the significance of environmental variables and LUC. *Sorghum halepense* was widely distributed worldwide through grain trade and forage introduction, except in Africa. Europe and North America provided most potential global suitable habitats (PGSHs) for *S. halepense* in cropland, grassland, and urban environments, representing 48.69%, 20.79%, and 84.82%, respectively. The future PGSHs of *S. halepense* increased continuously in the Northern Hemisphere, transferring to higher latitudes. Environmental variables were more significant than LUC in predicting the PGSHs of *S. halepense*. Future PGSHs of *S. halepense* are expected to increase, exacerbating the invasion risk through agricultural LUC. These results provide a basis for the early warning and prevention of *S. halepense* worldwide.

## 1. Introduction

Global changes, including international trade, climate change, and land-use change (LUC), are direct drivers of biological invasion [1,2]. The continuous increase in invasive alien plants (IAPs) is closely related to the globalization of trade, in which landscape gardening and the long-distance carriage of grass have been introduced in grassland and agriculture [3,4,5]. More than 500 species of IAPs are traded daily worldwide [6]. In the United States, 61% of the 1285 IAPs, including half of the state-regulated IAPs, are available via the plant trade [7]. Therefore, analyzing the invasive historical dynamics of IAPs is beneficial for understanding their invasion status and expansion trends. With global warming, IAPs could create new habitats at high elevations because of the suitable thermal conditions of climate change [8]. In addition, individuals first settle at low elevations and subsequently transfer to higher-elevation regions, causing LUCs in plant communities and increasing the possibility of IAPs [9,10]. Meanwhile, IAPs at low elevations are more likely to be affected by LUC [8]. These factors have a profound influence on native ecological communities, species interactions, and suitable ranges based on the ecology of invasive species [11]. The emergence and expansion of IAPs are notably increasing with climate warming and are expected to become more pronounced in association with LUC [12,13]. Therefore, to decrease the impact of IAPs, predicting the spatial dynamics of invasive species through an invasive risk assessment with species distribution models is beneficial for management to formulate relevant policies and measures for IAPs.

*Sorghum halepense* (Fabaceae) is among the ten most noxious and destructive global annual weeds [14]. Its exact native range is western Asia, the Mediterranean, and North Africa. However, broad distributions worldwide have recently been introduced as a consequence of crop seeds and forage cultivation, increasing invasion risk and threatening agriculture, economic development, and human health. The high density of *S. halepense* resulting from faster seed germination has led to the loss of plant diversity, drastically affecting cotton, soybean, and wheat production in Asia and North America [15,16,17]. *Sorghum halepense* seedlings, which have significant similarities with annual grains, when mixed with sorghum and maize plants have decrease fresh weight and increased crop yield losses in North America [15,18]. Mature *S. halepense* with a higher leaf area is a host of numerous pests, nematodes, and fungal pathogens for annual crops, including the sorghum midge (*Contarinia sorghicola*), *Meloidogyne incognita*, and leaf spot diseases (*Cercospora sorghi*) [19,20]. In addition, it can develop wider subterranean root systems that produce secondary metabolites, threatening medical plant development in Africa, such as *Plantago ovate* and *Ocimum basilicum* [21]. Furthermore, the pollen of *S. halepense* probably contributes to hay fever in North America, impacting human health. Additionally, *S. halepense* seeds are easily established in rangeland and agricultural fields, where they grow more effectively than C_4_ prairie plants, reducing native flora diversity [14]. *Sorghum halepense* extension has dramatically affected soil nutrition, soil temperature, and water content, leading to a C_3_ species decrease as well as a C_4_ group biomass increase [14]. Therefore, predicting the potential global suitable habitats (PGSHs) of *S. halepense* in ecological systems associated with future climate change and LUC has considerable practical value for agriculture and livelihoods.

Species distribution models (SDMs) are typically used to forecast the impacts of climate change on the potential spatial distribution of invasive species in ecology and biogeography [22]. SDMs combine species presence–absence data and related environmental conditions for distribution prediction, including generalized linear models (GLMs), maximum entropy (MaxEnt), random forest (RF), and CLIMEX [23,24,25,26]. Biomod2 is the most popular and well-constructed platform in the SDM community, composed of ten individual models (GLM, gradient boosting models (GBMs), RF, artificial neural network (ANN), generalized additive models (GAMs), flexible discriminant analysis (FDA), MaxEnt, classification tree analysis (CTA), surface range envelope (SRE), and multivariate adaptive regression splines (MARSs)) that can be randomly selected to produce ensemble models (EMs) [27,28]. With the high prediction accuracy achieved by minimizing the disadvantages of each individual model, EMs are more robust than individual models in the risk assessment of IAPs [29]. For instance, predicting the potential worldwide distribution of *Ambrosia* L. and the suitable areas of *Ageratum houstonianum*, *Chromolaena odorata*, *Hyptis suaveolens*, *Lantana camara*, *Mikania micrantha*, and *Parthenium hysterophorus* in Nepal has been widely attempted in biomod2 [30,31].

In the present study, we (a) reconstructed the historically invaded countries, analyzing the movement means and dispersal dynamics of *S. halepense* by collecting geographical information that detailed the longitude, latitude, and invasive countries as time elapsed; (b) predicted the PGSHs of *S. halepense* under climate change and LUC in the 2030s and 2050s, mainly extracting three land-use types (cropland, grassland, and urban areas); (c) analyzed the probability of suitable habitats for *S. halepense* among the three land-use types; and finally (d) screened the significant environmental variables for the PGSHs of *S. halepense.*

## 2. Results

### 2.1. Reconstruction of Historically Invaded Countries

**Figure 1:**
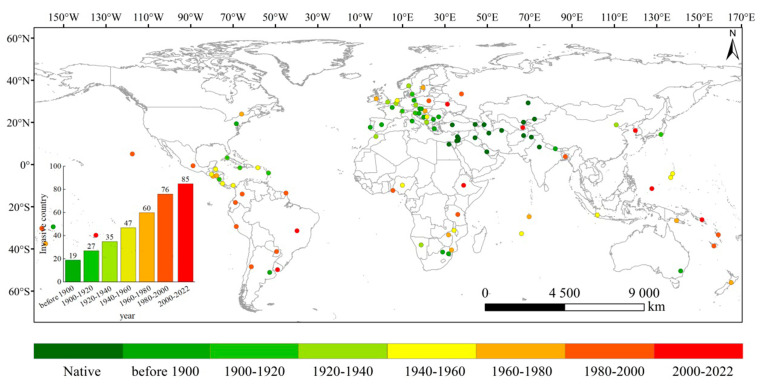
Reconstructed global countries invaded by *Sorghum halepense.* Eight time intervals are shown until 2022 from green to red, except for native countries. Temporal accumulation histograms of invaded countries are displayed in the bottom left corner.

The native regions of *S. halepense* were mainly distributed in southern Asia, eastern Africa, and southern Europe, following CABI and WOS (Figure 1; Excel S1). In Asia, the earliest introduction of Nepal was in 1805, and it was subsequently transferred from the native region of southern Asia to eastern Asia. As a result, it is widely distributed in western, southern, and eastern Asia. In 1900, *S. halepense* was introduced into the Mediterranean Sea in Europe and transferred to western Europe in 1880 [32]. *Sorghum halepense* reached northern Europe in the subsequent two decades and is now distributed throughout almost all of Europe. In the 1800s, *S. halepense* was discovered in the United States and spread to southern North America between 1900 and 2022 [33]. However, in South America, although it was first discovered in Argentina in the 1800s [34], it spread from southern North America and was introduced into new countries until the 1980s. In Africa, it spread to northeastern and northern Africa between the 1900s and 2022 [35]. It has been widely distributed throughout Oceania since *S. halepense* was introduced to Australia in 1871 [36]. In summary, *S. halepense* is now widespread in most countries worldwide, except Africa, which has a potential invasion risk.

### 2.2. Model Performance and Significant Environmental Variables

We calculated the model accuracy of ANN, CTA, FDA, GBM, GLM, MARS, MaxEnt, RF, and EM using ROC, TSS, and KAPPA values (Figure S1). The mean ROC of the eight models was 0.885, 0.943, 0.948, 0.960, 0.947, 0.952, 0.951, and 0.983, respectively (Table S1). The mean TSS of the eight individual models was 0.666, 0.818, 0.773, 0.809, 0.775, 0.778, 0.785, and 0.880, respectively. The mean KAPPA of the eight models was 0.685, 0.810, 0.768, 0.808, 0.777, 0.782, 0.778, and 0.880, respectively. The mean ROC, TSS, and KAPPA of the EM were 0.984, 0.851, and 0.856, respectively, which were higher than those of the individual models, indicating that the EM-predicted PGSHs of *S. halepense* were reliable.

We analyzed the significant environmental variables to predict the PGSHs of *S. halepense* using the EM, and the mean contributions are listed in Table 1. The precipitation of the coldest quarter (bio19, 0.317) was the most significant environmental variable, followed by the precipitation of the driest quarter (bio17, 0.108); the minimum temperature of the coldest month (bio6, 0.091); the annual precipitation (bio12, 0.085); the mean diurnal range (the mean of the monthly max temp-min temp) (bio2, 0.078); the max temperature of the warmest month (bio5, 0.067); precipitation seasonality (the coefficient of variation) (bio15, 0.048); and LUC (0.019). The response curves of the significant variables are shown in Figure S2. When the suitability probability achieved the maximum values, the survival probability of *S. halepense* was more reliable. Bio19 and bio17 were the most significant environmental variables for the predicted PGSHs of *S. halepense* in the three land-use types, achieving the approximate maximum values at 200 mm and 140 mm, respectively.

### 2.3. The PGSHs of S. halepense under Current and Future Climate Scenarios

**Figure 2:**
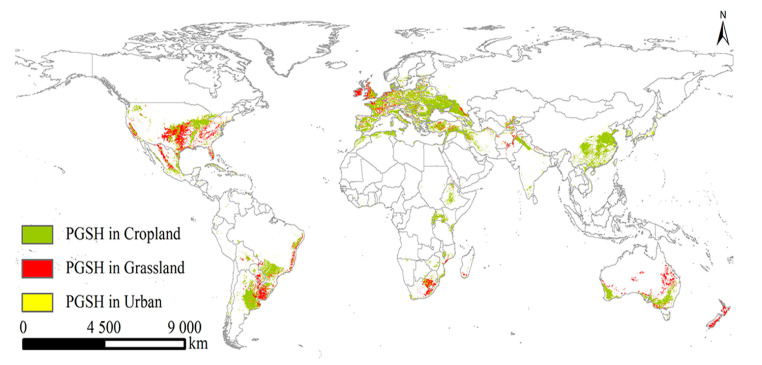
Potential global suitable habitats (PGSHs) of *Sorghum halepense* in three land-use types (cropland, grassland, and urban land) under the near-current climate scenario.

**Figure 3:**
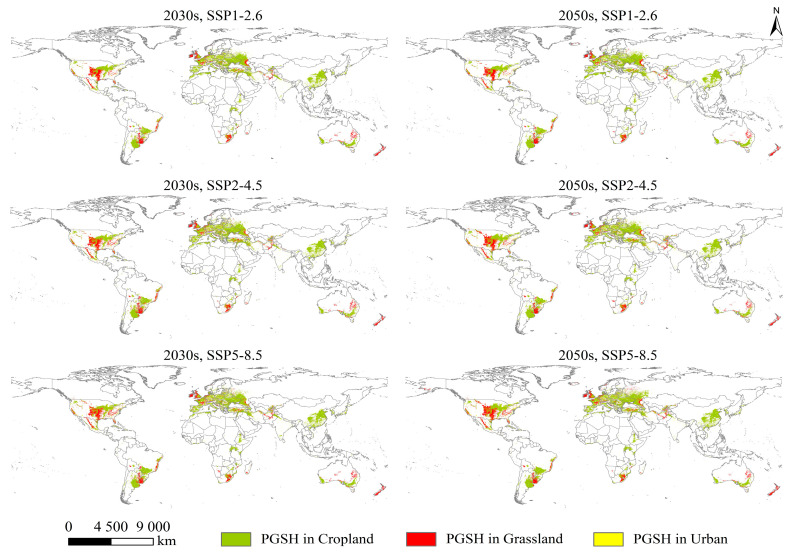
Potential global suitable habitats (PGSHs) of *Sorghum halepense* in three land-use types (cropland, grassland, and urban land) under SSP1-2.6, SSP2-4.5, and SSP5-8.5 in the 2030s and 2050s.

The PGSHs of *S. halepense* extracted from the three land-use types were mainly distributed in eastern, southeastern, central, and western Asia; western Europe; southern North America; southeastern South America; east-central, southwestern, and northern Africa; and southeastern and southwestern Oceania, as projected for near-current climate and future climate scenarios (Figure 2 and Figure 3). Compared with the near-current climate, the predicted global total cropland and urban areas would increase under future scenarios, yet the predicted total grassland area would decrease to a certain extent (Table 2). As a result, the PGSHs of *S. halepense* notably increased in the three land-use types, achieving a maximum under SSP5-8.5 in the 2030s and the 2050s.

In cropland, the PGSHs of *S. halepense* were located in eastern Asia (eastern Japan and South Korea); southeastern Asia (southern and eastern China); central Asia (southern and northern India, northeastern Pakistan, Afghanistan, southeastern Uzbekistan, western Tajikistan, and southern Kazakhstan); and western Asia (Iran, northern Iraq, northern Syria, and Turkey); nearly all western countries in Europe; southern North America (central and western United States, Mexico, Cuba, and the Dominican Republic); southeastern South America (southern and eastern Brazil, central Bolivia, Paraguay, eastern Argentina, and Chile); northern Africa (northern Morocco, northern Algeria, northern Tunisia); east-central Africa (Ethiopia and Kenya); southwestern Africa (Uganda, Malawi, Mozambique, Zimbabwe, and South Africa); and southeastern and southwestern Oceania (southeastern and southwestern Australia).

In grassland, the PGSHs of *S. halepense* were mainly located in central Asia (Pakistan, Afghanistan, Tajikistan, Uzbekistan, and Kazakhstan); western Asia (Turkey); western Europe (northern Mediterranean Sea and west coast); southern North America (the United States and Mexico); southeastern South America (central Bolivia, eastern and southwestern Brazil, Paraguay, Uruguay, and northeastern Argentina); southern Africa (Ethiopia, eastern Mozambique, Lesotho, southern Madagascar, and South Africa); and southeastern and southwestern Oceania (Australia and New Zealand). However, in urban areas, the PGSHs of *S. halepense* were broadly distributed worldwide.

The suitable areas for *S. halepense* in cropland were mainly located in Europe under the near-current climate scenario, spread over approximately 311.95 × 10^4^ km, accounting for 33.83% of the PGSHs of *S. halepense*, followed by Asia (228.21 × 10^4^, 24.75%); South America (127.91 × 10^4^, 13.87%); North America (117.19 × 10^4^, 12.71%); Africa (79.29 × 10^4^, 8.60%); and Oceania (54.21 × 10^4^, 5.88%). In the 2030s, future suitable cropland areas achieved the maximum extent under SSP2-4.5 in Europe, at approximately 379.84 × 10^4^ km, accounting for 33.83% of the PGSHs of *S. halepense*, followed by Asia (249.93 × 10^4^, 24.35%); South America (151.74 × 10^4^, 14.38%); North America (147.60 × 10^4^, 14.38%); Africa (85.07 × 10^4^, 8.29%); and Oceania (55.69 × 10^4^, 5.43%). In the 2050s, future suitable cropland areas for *S. halepense* were mainly distributed in Europe, spread over approximately 407.34 × 10^4^ km, accounting for 36.57% of the PGSHs of *S. halepense*, followed by Asia (254.32 × 10^4^, 22.83%); North America (160.65 × 10^4^, 14.42%); South America (148.04 × 10^4^, 13.29%); Africa (87.94 × 10^4^, 7.89%); and Oceania (52.28 × 10^4^, 4.69%).

Suitable areas for *S. halepense* in grassland were mainly located in North America under the near-current climate scenario, spread over approximately 95.63 × 10^4^ km, accounting for 34.10% of the PGSHs of *S. halepense*, followed by Europe (55.47 × 10^4^, 19.78%); South America (46.70 × 10^4^, 16.65%); Oceania (43.16 × 10^4^, 15.39%); Africa (19.51 × 10^4^, 6.96%); and Asia (18.2 × 10^4^, 6.49%). In the 2030s, future suitable grassland areas achieved the maximum extent under SSP5-8.5 in North America at approximately 116.18 × 10^4^ km, accounting for 36.84% of the PGSHs of *S. halepense*, followed by Europe (66.83 × 10^4^, 21.19%); South America (43.55 × 10^4^, 13.81%); Oceania (39.18 × 10^4^, 12.42%); Asia (25.53 × 10^4^, 8.09%); and Africa (22.30 × 10^4^, 7.07%). In the 2050s, future suitable grassland areas for *S. halepense* were mainly distributed in North America, spread over approximately 131.39 × 10^4^ km, accounting for 39.27% of the PGSHs of *S. halepense*, followed by Europe (81.90 × 10^4^, 24.48%); South America (39.69 × 10^4^, 11.86%); Oceania (33.83 × 10^4^, 10.11%); Asia (26.96 × 10^4^, 8.06%); and Africa (18.82 × 10^4^, 5.62%).

Suitable areas for *S. halepense* in urban environments were mainly located in Europe under the near-current climate scenario, spread over approximately 14.98 × 10^4^ km, accounting for 29.71% of the PGSHs of *S. halepense*, followed by North America (14.18 × 10^4^, 28.12%); Asia (13.00 × 10^4^, 25.78%); South America (3.02 × 10^4^, 5.99%); Africa (2.50 × 10^4^, 4.96%); and Oceania (1.24 × 10^4^, 2.46%). In the 2030s, future suitable urban areas achieved the maximum extent under SSP5-8.5 in North America at approximately 25.01 × 10^4^ km, accounting for 31.73% of the PGSHs of *S. halepense*, followed by Europe (20.47 × 10^4^, 25.97%); Asia (20.05 × 10^4^, 25.44%); Africa (4.81 × 10^4^, 6.10%); South America (4.06 × 10^4^, 5.15%); and Oceania (2.46 × 10^4^, 3.12%). In the 2050s, future suitable urban areas for *S. halepense* were mainly distributed in North America, spread over approximately 34.10 × 10^4^ km, accounting for 35.62% of the PGSHs of *S. halepense*, followed by Europe (24.43 × 10^4^, 25.52%); Asia (21.42 × 10^4^, 22.37%); Africa (5.65 × 10^4^, 5.90%); South America (4.17 × 10^4^, 4.36%); and Oceania (3.67 × 10^4^, 3.83%).

### 2.4. Changes in PGSHs of S. halepense

**Figure 4:**
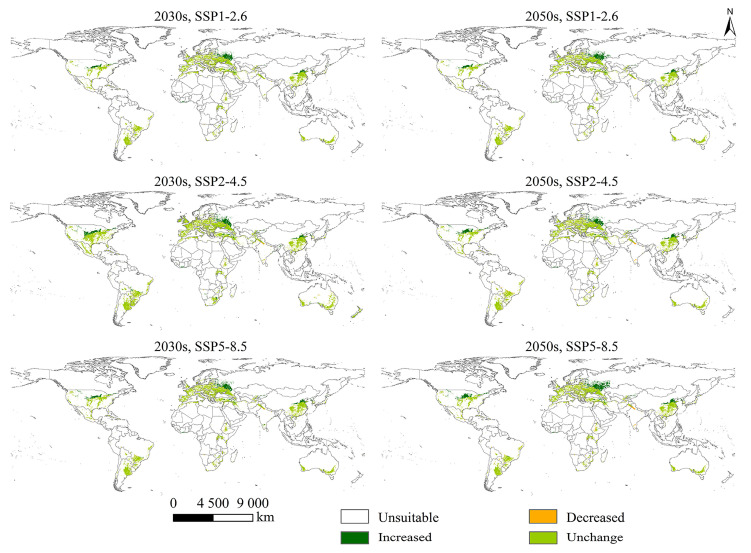
Changes in suitable cropland area for *Sorghum halepense* from different future scenarios (SSP1-2.6, SSP2-4.5, and SSP5-8.5) in the 2030s and 2050s compared to the near-current climate scenario.

Future increased cropland habitats were virtually all distributed in the North Hemisphere, including eastern, central, and western Asia (China, Iran, Kazakhstan, and Turkey); southwestern Europe (Russia and Belarus); and central North America (Canada and the United States), as well as parts of South America (Brazil) and Africa (Cote d’ Ivoire) (Figure 4). The suitable area achieved its maximum extent under SSP5-8.5 in the 2050s, at approximately 172.09 × 10^4^ km^2^. Future decreased cropland habitats were mainly distributed in central Asia (Pakistan and India), as well as sporadically in southern North America (Mexico); central South America (Argentina, Bolivia, Brazil, and Paraguay); and eastern and central Africa (Ethiopia, Congo, and Zimbabwe). The suitable area achieved its minimum extent under SSP5-8.5 in the 2050s, at approximately 29.21 × 10^4^ km^2^.

Future increased grassland habitats were virtually all distributed in the Northern Hemisphere, including central and western Asia (Afghanistan, Kyrgyzstan, Tajikistan, and Turkey); southern and southwestern Europe (Austria, Norway, Russia, San Marino, Slovenia, Switzerland, and the United Kingdom); and central North America (the United States), as well as parts of southern Africa (Namibia and South Africa) (Figure S3). The suitable area achieved the maximum extent under SSP5-8.5 in the 2050s at approximately 72.58 × 10^4^ km^2^. Future decreased grassland habitats were virtually all distributed in eastern, central, and western Oceania (Australia). The suitable area achieved the minimum extent under SSP5-8.5 in the 2050s at approximately 12.64 × 10^4^ km^2^.

In the future scenarios, there was no decrease in suitable urban habitats, which were distributed in the Northern Hemisphere, including eastern Asia (China), southern Europe (Russia), and central North America (the United States) (Figure S4). The suitable area achieved its maximum extent under SSP5-8.5 in the 2050s, at approximately 3.62 × 10^4^ km^2^.

### 2.5. Trend of Suitabillity Probability for S. halepense according to Latitudinal Gradient

**Figure 5:**
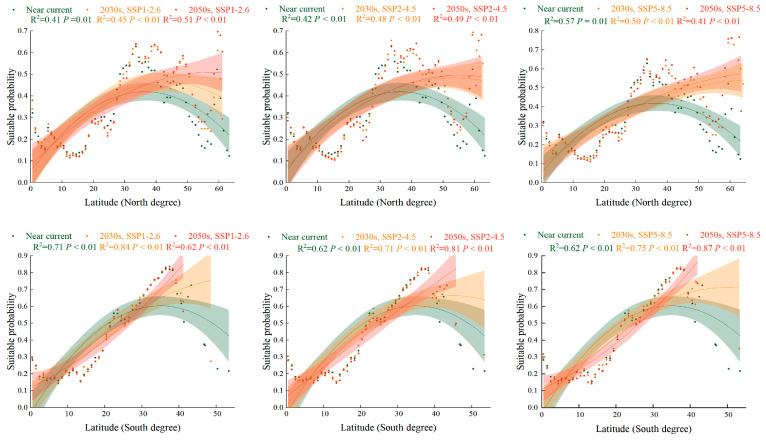
The PGSHs of *Sorghum halepense* in cropland for the Southern and Northern Hemispheres in the near-current climate scenario and under SSP1-2.6, SSP2-4.5, and SSP5-8.5 in the 2030s and 2050s. Green color means the near near-current climate scenario, orange color means the different climate scenario in the 2030s, and red is in the 2050s. For each group, we added 95% confidence bands via binomial fitting.

Compared with the near-current climate, the PGSHs of *S. halepense* in croplands tended to have high latitudinal gradients with a higher suitability probability under SSP1-2.6, SSP2-4.5, and SSP5-8.5 in the 2030s and 2050s (Figure 5). In the Northern Hemisphere, the suitable area for *S. halepense* in croplands was positioned at 28° N–39° N under a near-current climate, while it increased to 31° N–42° N, 35° N–50° N, and 58° N–61° N under future scenarios. In the Southern Hemisphere, the suitable area for *S. halepense* in croplands was positioned at 22° S–43° S under a near-current climate, while remaining firm at 22° S–42° S in future scenarios.

**Figure 6:**
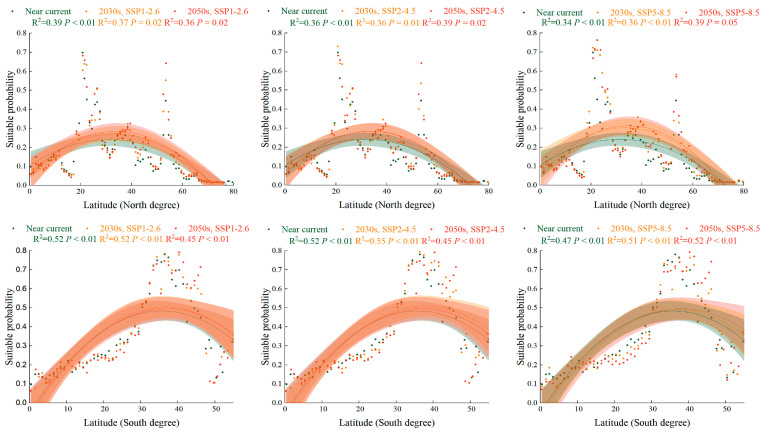
The PGSHs of *Sorghum halepense* in grassland for the Southern and Northern Hemispheres in the near-current climate scenario and under SSP1-2.6, SSP2-4.5, and SSP5-8.5 in the 2030s and 2050s. Green color means the near near-current climate scenario, orange color means the different climate scenario in the 2030s, and red is in the 2050s. For each group, we added 95% confidence bands via binomial fitting.

The global suitability probability of *S. halepense* in grasslands increased slightly for higher latitudinal gradients under SSP1-2.6, SSP2-4.5, and SSP5-8.5 in the 2030s and 2050s compared with the near-current climate scenario (Figure 6). In the Northern Hemisphere, the suitable area for *S. halepense* in grasslands was positioned at 20° N under the near-current climate, while it increased to 20° N–26° N under future scenarios. In the Southern Hemisphere, the suitable area for *S. halepense* in grasslands was positioned at 30° S–41° S under the near-current climate, while it increased to 31° S–46° S under future scenarios.

**Figure 7:**
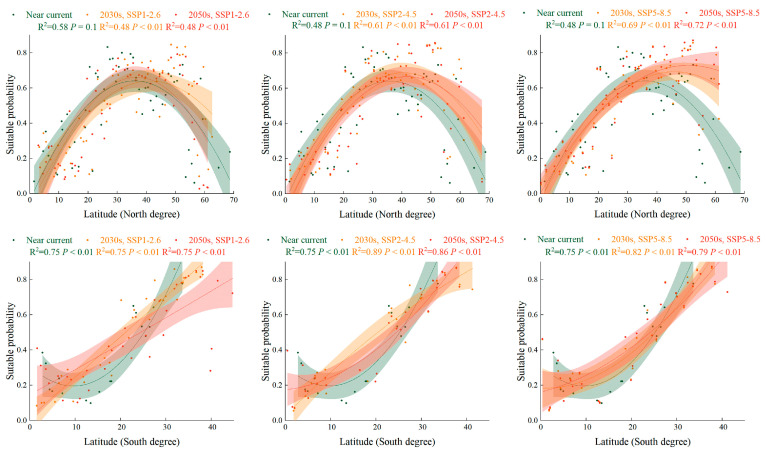
The PGSHs of *Sorghum halepense* in urban land for the Southern and Northern Hemispheres in the near-current climate scenario and under SSP1-2.6, SSP2-4.5, and SSP5-8.5 in the 2030s and 2050s. Green color means the near near-current climate scenario, orange color means the different climate scenario in the 2030s, and red is in the 2050s. For each group, we added 95% confidence bands via binomial fitting.

The global suitability probability of *S. halepense* in urban areas increased dramatically for higher latitudinal gradients under SSP1-2.6, SSP2-4.5, and SSP5-8.5 in the 2030s and the 2050s compared with the near-current climate scenario (Figure 7). In the Northern Hemisphere, the suitable area for *S. halepense* in urban land was positioned at 20° N–52° N under a near-current climate, while it increased to 19° N–61° N under future scenarios. In the Southern Hemisphere, the suitable area for *S. halepense* in urban land was positioned at 22° S–33° S under the near-current climate, while it increased to 26° S–41° S under future scenarios.

## 3. Discussion

*Sorghum halepense*, with its high reproduction rate and quick dispersal, is a well-known IAP worldwide that has a negative influence on crops, such as cotton, soybean, and wheat, in croplands and grasslands [15,16,17]. This study analyzed the dispersal modes and distribution dynamics of *S. halepense* according to reconstructions of the global countries historically invaded by *S. halepense* mixed in with crop seeds and introduced as forage based on CABI, GBIF, and WOS [5]. The expansion of IAPs is driven by climate change and possibly associated with LUC [13]. The impacts of climate change, LUC, and their interactions on habitat suitability for IAPs have been broadly investigated; however, few studies have examined how future LUC-associated climate change might affect IAPs [37,38]. This is the first study to explore the joint effect of LUC and climate change on the PGSHs of *S. halepense* under the near-current climate and future climate scenarios using the biomod2 model, which could be a vital premise for the management and prevention of IAPs.

### 3.1. Historical Invasion Reconstruction

The globalization of trade has facilitated the invasion of IAPs by intentionally moving them away from their native range for commercial purposes or accidentally introducing them into new environments [39]. The future dynamics of IAPs remain unclear and will be associated with socioeconomic changes and increased human populations, which are difficult to predict [40]. *Sorghum halepense* has been either introduced as a potential fodder or imported and exported mixed with grains, which are vital factors for rapid invasion via global trade [41]. Early in the 1800s, as a potential fodder plant and pasture grass, it was probably introduced from Turkey to South Carolina and Argentina and subsequently introduced to Australia and the southeastern United States, California, and New Mexico by farmers in the middle of the 1800s [39]. Our results showed that the countries invaded by *S. halepense* are broadly distributed worldwide, except for central Asia (Myanmar and Mongolia); northern Europe (Byelarus, Latvia, Lithuania, and Estonia); northern North America (Greenland); northern South America (Venezuela, Guyana, and Surname); and central, western, and northern Africa (Chad, Mali, and Libya). Furthermore, global crop reproduction and pasturing livestock, which are vulnerable to population growth and climate change, are likely to affect the invasion of *S. halepense* [42]. How socioeconomic drivers will impact the future distribution of IAPs is unknown, but the influence of socioeconomic dynamics could facilitate the invasion and establishment of IAPs in new environments depending on climate change and LUC [40].

### 3.2. Impact of Climate Change and LUC on Suitable Habitats

A comparison of the effects of climate change and LUC on IAPs has shown that the impact of LUC on the suitable ranges for invasive plants is weaker than that of climate change [43]. Our results showed that the contribution of LUC to predicting the distribution of *S. halepense* was smaller than that of climate change (Table 2). Climate change plays an important role in the distribution of IAPs at larger scales, whereas LUC has a stronger influence on variables at smaller scales [44]. Considering the invasion of *S. halepense* in croplands and grasslands, we explored the combined effects of climate change and LUC on the distribution of invasive plants at the global scale. With rising temperatures and elevated CO_2_ emissions, the PGSHs of *S. halepense* will increase in the future and transfer to higher altitudes in cropland, grassland, and urban areas. Climate change may facilitate plant transfer along elevational gradients. IAPs respond faster to climate change than native plants [45,46]. Warming occurs in mid-to-high latitude regions; therefore, more adaptable plants are expected to exhibit elevated shifts [39].

Climate change may expand the range of IAPs by changing their traits, such as greater high-temperature tolerance, better adaptable growth with elevated CO_2_ emissions, and latitude dependence [47,48]. Temperature is a vital factor that restricts plant growth and reproduction and is closely correlated with latitude [2]. Therefore, IAPs are expected to be transferred to higher latitudes with climate warming [49]. Our results showed that the future PGSHs of *S. halepense* in the three land-use types transferred to higher latitudes under the future scenarios. In contrast, future changes in precipitation are more variable than those in temperature under elevated emission scenarios [50]. *Sorghum halepense*, with its high adaptability in new environments, could tolerate a maximum temperature of 35 °C and temperatures as low as −26 °C at the coldest time of year. In addition, the mean annual precipitation that fits with *S. halepense* growth ranges widely from 300 to 2000 mm. The odds of future climate change that will cause extreme climates are significantly increasing. Our results showed that the future PGSHs of *S. halepense* are expected to increase in the Northern Hemisphere, achieving their maximum extent under SSP5-8.5 in the 2050s (Table 2). This is similar to other IAPs, whose potentially suitable habitats will change in response to climate change. For instance, the suitable global habitats of three ragweeds (*Ambrosia artemisiifolia*, *A. psilostachya*, and *A. trifida*) are expected to increase by the 2050s [30].

With future climate warming, many regions will become increasingly hot, leading to frequent droughts that will cause physiological stress and mortality in invasive and native plants, which is expected to cause grassland degradation [51]. However, IAPs’ responses to temperature, precipitation, and landscape changes outcompete those of native plants; therefore, invasive plants will become dominant [52]. Previous studies have shown that *S. halepense* has a climate suitability of 50–90% in plains and grasslands in the United States, which poses a potential risk of transforming native grasslands into invaded prairies [53]. Our results showed that the global grassland area projected for future LUC scenarios decreased to some extent, yet the invasive area of *S. halepense* progressively increased (Table 2; Figure 3). In addition, cropland expansion has been gradually increasing in tropical areas since 2000 and is closely correlated with urban expansion [54]. To satisfy the housing demands of an increasing urban population, urban land is expected to increase dramatically in the future. More than 60% of global croplands are distributed near urban land; therefore, urban expansion is expected to be transferred from croplands [55,56]. Hence, the future PGSHs of *S. halepense* in cropland and urban areas will increase northward, and more suitable areas will be located in eastern China, western Europe, and northern North America under future climate scenarios (Table 2; Figure 3). Similarly, future increases in the PGSHs of *S. halepense* in grasslands will be located in western Europe and northern North America.

### 3.3. Prevention and Control

The unintentional introduction of *S. halepense* via the global grain trade and its intentional introduction as a forage crop have been key to *S. halepense*’s spread [5]. Our results showed that future increases in the PGSHs of *S. halepense* in the three land-use types will mainly be located in eastern China, Turkey, Iran, Afghanistan, Tajikistan, and southern Kazakhstan in Asia; Austria, Norway, San Marino, Slovenia, Switzerland, the United Kingdom, western Russia, and Belarus in Europe; the northern United States and southern Canada in North America; southern Brazil in South America; and southern Cote d’ Ivoire, South Africa, Ghana, and Ethiopia in Africa. For newly invaded countries such as San Marino, Belarus, Cote d’ Ivoire, and Ghana, identifying early warnings of *S. halepense* through risk assessment is important. However, for the newly invaded regions, customs quarantine controls in each country will play a crucial role in resisting the expansion and spread of *S. halepense*. Furthermore, regarding the invasion and establishment of *S. halepense*, there are a few measures to control population outbreaks, such as chemical control and integrated pest management (IPM). Selective herbicides may be effective against *S. halepense*, including quizalofop, nicosulfuron, and glyphosate, achieving 88–97% effectiveness [57]. In addition, the pre-sowing treatment of soybean and sorghum with glyphosate, Merlin, and Stromp is a vital method for controlling *S. halepense* [58,59,60].

## 4. Materials and Methods

### 4.1. Occurrence Data and Reconstructed Historically Invaded Countries

The global occurrences of *S. halepense* were collected from the Global Biodiversity Information Facility (GBIF: accessed on 13 December 2022, https://www.gbif.org/); aBarcode of Life Data Systems (BOLD: accessed on 19 December 2022, http://boldsystems.org/); the Southwest Environmental Information Network (SNIet: accessed on 6 December 2022, http://swbiodiversity.org); the Ministry of Agriculture and Rural Affairs of the People’s Republic of China (accessed on 7 December 2022, http://www.moa.gov.cn/); some published literature on Web of Science (WOS: accessed on 6 December 2022, https://www.webofscience.com/); and field sampling in China. A total of 32,972 occurrences of *S. halepense* were found in 124 countries (including regions, as stated below) (Supplementary Excel). To avoid a sampling bias in constructing the species distribution model, occurrences of *S. halepense* were filtered using ENMTools, retaining only one occurrence in a 10 × 10 km raster [61]. The final occurrence data comprised 21,443 occurrences of *S. halepense* (Figure S5).

To reconstruct the countries historically invaded by *S. halepense*, we first removed 21 countries, including 18 countries that had no detailed geographical information on the occurrence of *S. halepense* and three countries without recorded databases and literature, compared with countries recorded by the Centre for Agriculture and Biosciences International (CABI: accessed on 21 February 2023, https://www.cabi.org/) and European and Mediterranean Plant Protection Organization (EPPO: accessed on 21 February 2023, https://www.eppo.int/) based on GBIF and WOS. The earliest recorded time for field sampling and the literature review was assumed to be time of invasion in each country. We confirmed 18 countries to which *S. halepense* was native based on CABI and EPPO. The invaded countries recorded before 1900 were regarded as one category, and other invaded countries from 1900 to 2022 with an interval of 20 years were used to reconstruct the global spatio-temporal dynamics of *S. halepense* invasion. In addition, we calculated the variation dynamics of the number of invasive countries over time and analyzed the invasive trends of *S. halepense*. Geographical observations were set to the World Geodetic System 1984 geographic coordinate system and imported to ArcGIS 10.8. We presented the capital of each country invaded by *S. halepense* in ArcGIS.

### 4.2. Land-Use Harmonization Data

Land-use harmonization data were used to examine how changes in the PGSHs of *S. halepense* may be affected by LUC. The annual datasets for 2015–2100 with a 1 km resolution estimated the fractional land-use patterns, underlying land-use transitions, and key agricultural management information [62]. In addition, we obtained historical reconstructions of land-use in the annual fraction state layers for 2015, representing near-current climate data. The future harmonized land-use dataset covered 2040 and 2060 to match future periods of the baseline, including SSP1-2.6, built with IMAGE; SSP2-4.5, built with MESSAGE-GLOBIOM; and SSP5-8.5, built with REMIND-MAGPIE [63]. The harmonized land classifications considered were mainly cropland, grassland, urban areas, and others, which could be transferred from one land-use type to another (Figure S6).

We predicted the potentially suitable and unsuitable habitats for *S. halepense* during the near-current and future periods, quantifying the proportion of land use for each classification in every grid cell. In addition, the predicted PGSHs of *S. halepense* under the near-current and future climate scenarios were mainly located in three land-use types: cropland, grassland, and urban areas. Therefore, by analyzing the decreased, increased, or unchanged PGSHs for *S. halepense* towards the middle of the century, we could examine the response to climate change and LUC from the near-current climate to future scenarios.

### 4.3. Climate Data

We downloaded 19 historical environmental variables from 1970 to 2000 and the future period (2021–2040 and 2041–2060) at a 5 min spatial resolution from WorldClim v2.1 [64]. Future data were based on BCC-CSM2-MR global climate models (GCMs) for four shared socio-economic pathways (SSPs): SSP1-2.6, SSP2-4.5, and SSP5-8.5. SSP1-2.6 is regarded as the future ideal scenario, in that 1.5 °C global warming is avoided, while the extreme scenario SSP5-8.5 is more likely to occur [65,66]. Hence, climate change and LUC analyses mainly focused on the emission scenarios SSP2-4.5 and SSP5-8.5 to estimate the spatial dynamics and PGSHs of *S. halepense*. In addition, strong correlations between environmental variables could lead to multicollinearity [67]. We used ENMTools to examine the variable correlations (|r| ≥ 0.8) and removed variables that were strongly correlated (Figure S7). Finally, we retained seven significant environmental variables for *S. halepense* (Table 2).

### 4.4. Model Construction and Evaluation

We predicted the PGSHs of *S. halepense* under the near-current climate and future scenarios based on occurrence records and environmental variables that included climate data and harmonized land-use data using eight individual models, including GLM, GBM, RF, Maxnet, CTA, ANN, FDA, and MARS in the biomod2 4.2.3 package in Rstudio [68]. The training data randomly used 75% of the occurrence data, and the remaining 25% was selected as the testing dataset, with five model replicates. Ten thousand selected global pseudoabsence points were randomly replicated once to run the models. Finally, 40 models were reconstructed, and their performances were evaluated using the test dataset. In the present study, we selected a single model in which the true skill statistic (TSS) was higher than 0.8 and the area under the receiver operating characteristic (ROC) curve (AUC) was higher than 0.9 from 40 models to construct an EM predicting the PGSHs of *S. halepense* under the near-current climate and future scenarios.

TSS, AUC, and KAPPA were used to estimate the model performance [69]. TSS is an independent threshold calculated as sensitivity and specificity-1 ranging from −1 to +1 [70]. A value closer to +1 indicates excellent performance, and zero or less indicates worse performance than random [70]. The AUC is a metric that varies in true-positive and -negative rates and ranges from 0 to 1, with values closer to 1 indicating more perfect discrimination. KAPPA ranges from −1 to +1, and values closer to +1 indicate perfect performance [70]. The probability (P) of the presence of *S. halepense* was generated in the ASCII raster and ranged from 0 to 1 in the model results. We classified the PGSHs of *S. halepense* into two categories based on the maximum KAPPA value: unsuitable habitats (0 < *p* ≤ 0.37) and suitable habitats (0.37 < *p* ≤ 1).

## 5. Conclusions

Our study was the first to reconstruct the countries historically invaded by *S. halepense* and explore how climate change and LUC will influence the PGSHs of *S. halepense* in the three land-use types (croplands, grasslands, and urban areas). *Sorghum halepense* is widely found worldwide except in Africa due to the import and export trade and the introduction of fodder grass species. Our results found that environmental variables were more significant than LUC in predicting the PGSHs of *S. halepense*. In addition, future croplands and urban areas are expected to increase continuously, while grassland areas are expected to decrease. The future PGSHs of *S. halepense* in the three land-use types are expected to increase continuously in the Northern Hemisphere and transfer to higher latitudes with climate warming. Furthermore, new invaded countries, such as San Marino, Belarus, Cote d’Ivoire, and Ghana, need risk assessments of *S. halepense* to prevent its introduction. The threatened regions in the six continents need to increase plant quarantines at customs facilities in each country. The reconstruction of the countries historically invaded by *S. halepense* and the predicted PGSHs of *S. halepense* will help provide a more reliable risk assessment.

## Figures and Tables

**Table 1:** Significant screened environmental variables and contribution importance.

Variable	Description	Unit	Importance
bio2	Mean diurnal range (mean of monthly max temp-min temp)	°C	0.078
bio5	Max temperature of warmest month	°C	0.067
bio6	Min temperature of coldest month	°C	0.091
bio12	Annual precipitation	mm	0.085
bio15	Precipitation seasonality (coefficient of variation×1)	-	0.048
bio17	Precipitation of driest quarter	mm	0.108
bio19	Precipitation of coldest quarter	mm	0.317
LUC	Land-use change		0.019

**Table 2:** Potential global suitable habitats (PGSHs) of *Sorghum halepense* in three land-use types (cropland, grassland, and urban land) for the near-current climate and different future scenarios.

Land Use (×104 km)	Cropland			Grassland			Urban		
	Suitable Area	Total Area		Suitable Area	Total Area		Suitable Area	Total Area	
Near-current	921.99	2177.42	42.34%	280.41	1675.80	16.73%	50.42	61.89	81.47%
2030s, SSP1-2.6	1026.27	2150.03	47.73%	297.00	1555.53	19.09%	75.24	89.92	83.67%
2030s, SSP2-4.5	1073.00	2279.08	47.08%	317.87	1638.99	19.39%	72.92	87.09	83.73%
2030s, SSP5-8.5	1079.24	2340.77	46.11%	315.38	1613.94	19.54%	78.81	93.78	84.04%
2050s, SSP1-2.6	1060.59	2178.33	48.69%	281.28	1448.65	19.42%	85.00	101.57	83.69%
2050s, SSP2-4.5	1091.27	2345.28	46.53%	315.51	1577.10	20.01%	82.24	99.20	82.90%
2050s, SSP5-8.5	1113.91	2377.42	46.85%	334.58	1609.21	20.79%	95.63	112.74	84.82%

## Data Availability

The data presented in this study are available in this article.

## Supplementary Materials

The following supporting information can be downloaded at: https://www.mdpi.com/article/10.3390/plants12173128/s1, Figure S1: Single model performance with ROC and TSS values, Figure S2: Significant environmental variables for predicting the potential global suitable habitats (PGSHs) of *Sorghum halepense*, Figure S3: Changes in suitable grassland areas for *Sorghum halepense* under different future scenarios (SSP1-2.6, SSP2-4.5, and SSP5-8.5) in the 2030s and 2050s compared to the near-current climate, Figure S4: Changes in suitable urban areas for *Sorghum halepense* under different future scenarios (SSP1-2.6, SSP2-4.5, and SSP5-8.5) in the 2030s and 2050s compared to the near-current climate, Figure S5: Global distribution occurrences of *Sorghum halepense*, Figure S6: Global land-use transitions under future scenarios. Each arrow represents a change from a specific land-use type to another land-use type. (A) the transitions from SSP1-2.6 in the 2030s to the near-current climate, (B) the transitions from SSP1-2.6 in the 2050s to SSP1-2.6 in the 2030s, (C) the transitions from SSP2-4.5 in the 2030s to the near-current climate, (D) the transitions from SSP2-4.5 in the 2050s to SSP2-4.5 in the 2030s, (E) the transitions from SSP5-8.5 in the 2030s to the near-current climate, (F) the transitions from SSP5-8.5 in the 2050s to SSP5-8.5 in the 2030s, Figure S7: Correlation of twenty environmental variables via ENMTools, Table S1: The mean ROC, TSS, and KAPPA values of eight individual models and the EM, Table S2: Environmental variables projected to predict the potential global suitable habitats (PGSHs) of *Sorghum halepense*, Excel S1: Countries historically invaded by *Sorghum halepense*.
