# Supplementary material for: Reconstructed Global Invasion and Spatio-Temporal Distribution Pattern Dynamics of Sorghum halepense under Climate and Land-Use Change
Source: Plants (Basel). 2023 Aug 31;12(17):3128. doi: 10.3390/plants12173128 (PMC10489930; doi:10.3390/plants12173128)
Supplement: Supplementary file 1 [file plants-12-03128-s001.zip › Supplemental material.pdf]

Supplemental materials

Supplemental figure

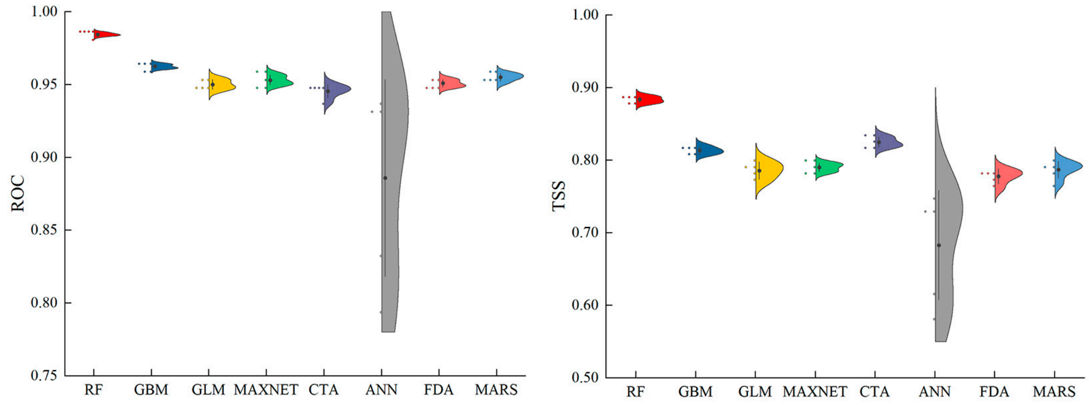

Figure S1. Single model performance with ROC and TSS values.

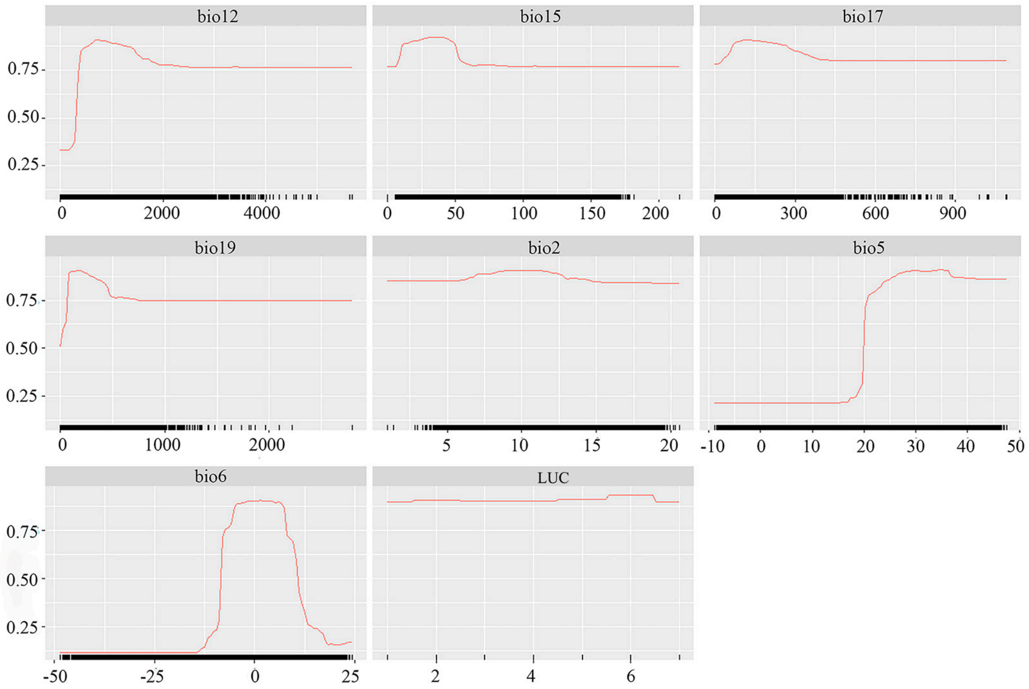

Figure S2. Significant environmental variables to predict the potential global suitable habitats (PGSH) of *Sorghum halepense.*

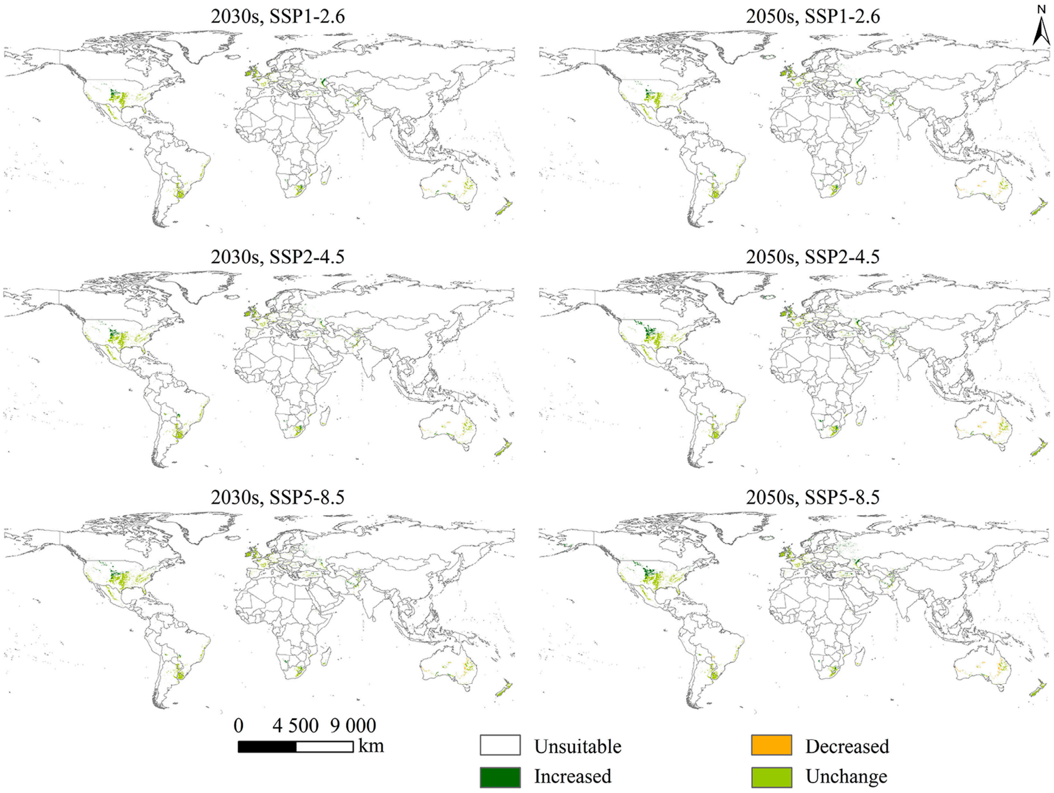

Figure S3. Changes in suitable grassland area for *Sorghum halepense* from different future scenarios (SSP1-2.6, SSP2-4.5, and SSP5-8.5) in the 2030s and 2050s to the near current climate.

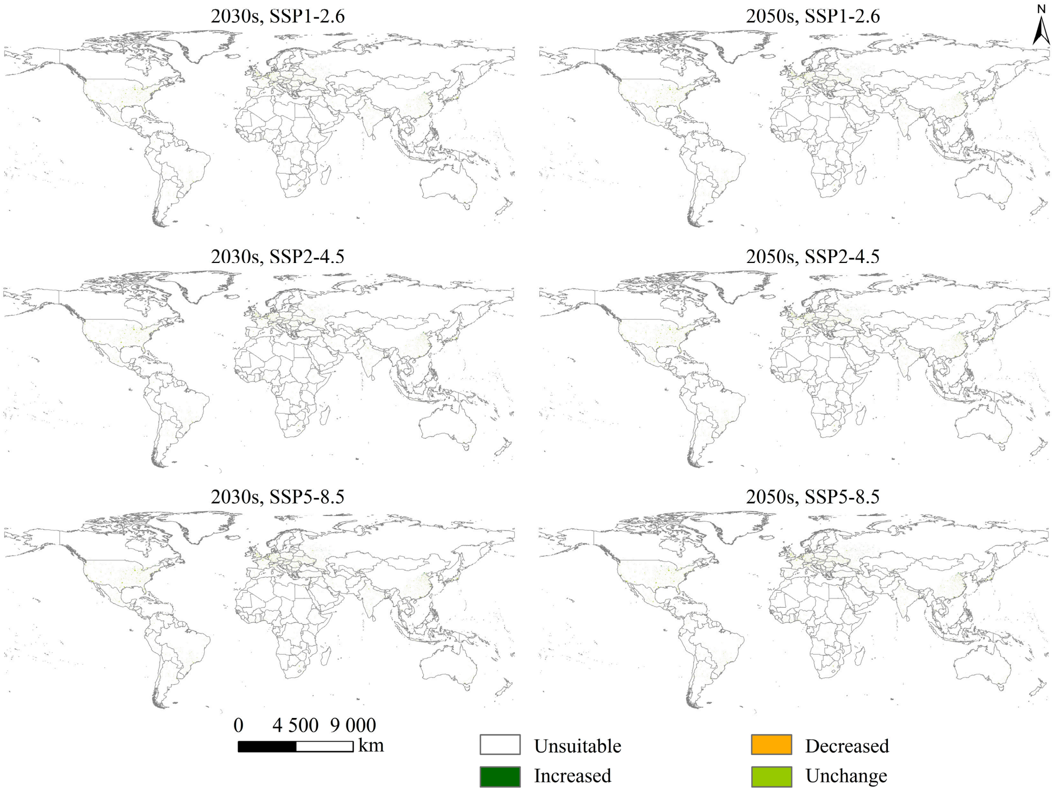

Figure S4. Changes in suitable urban area for *Sorghum halepense* from different future scenarios

(SSP1-2.6, SSP2-4.5, and SSP5-8.5) in the 2030s and 2050s to the near current climate.

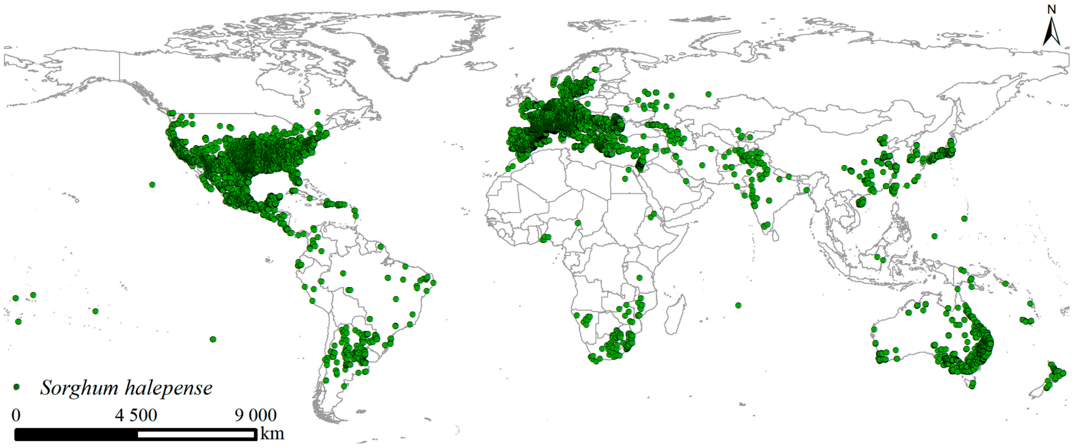

Figure S5. Global distribution occurrences of *Sorghum halepense.*

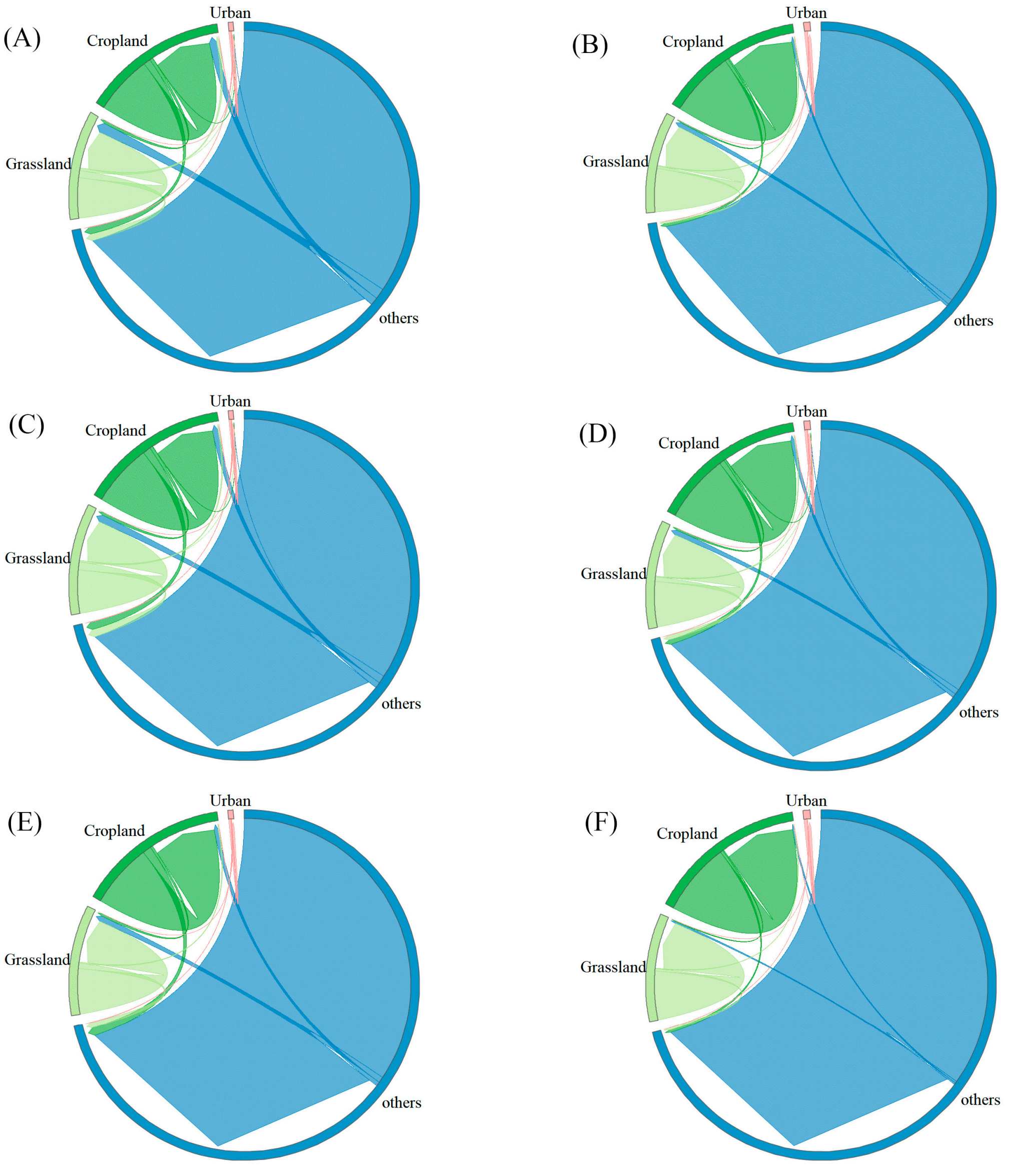

Figure S6. Global land-use transitions by future scenarios. Each arrow represents from a specific land-use type to other land-use type. (A) the transitions from under SSP1-2.6 in the 2030s to the near current climate, (B) the transitions from under SSP1-2.6 in the 2050s to the SSP1-2.6 in the 2030s, (C) the transitions from under SSP2-4.5 in the 2030s to the near current climate, (D) the transitions from under SSP2-4.5 in the 2050s to the SSP2-4.5 in the 2030s, (E) the transitions from under SSP5-8.5 in the 2030s to the near current climate, (F) the transitions from under SSP5-8.5 in the 2050s to the SSP5-8.5 in the 2030s.

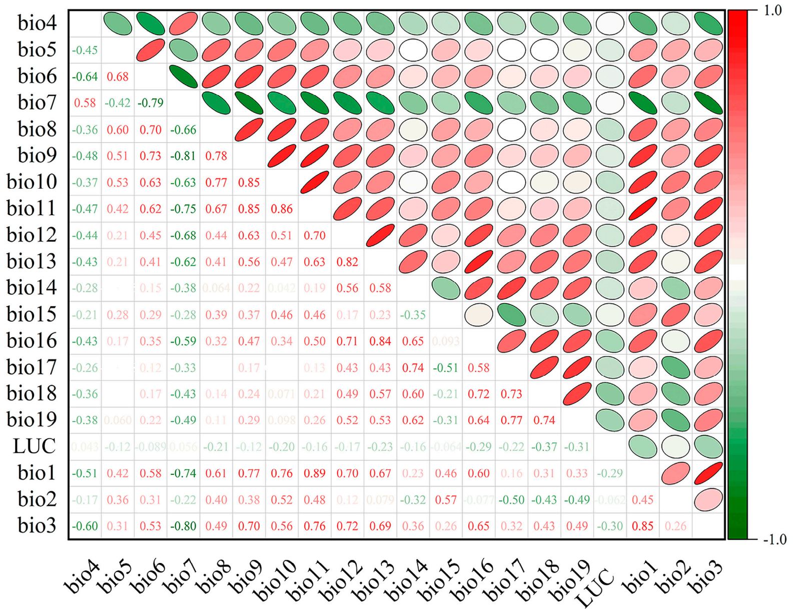

Figure S7. Correlation of twenty environmental variables via ENMTools.

Supplemental table

Table S1 The mean ROC, TSS, and KAPPA values of eight single model and EM.

model	mean ROC	mean TSS	mean KAPPA
ANN	0.885	0.666	0.685
CTA	0.943	0.818	0.810
FDA	0.948	0.773	0.768
GBM	0.960	0.809	0.808
GLM	0.947	0.775	0.777
MARS	0.952	0.778	0.782
MaxEnT	0.951	0.785	0.778
RF	0.983	0.880	0.880
EM	0.984	0.851	0.856

Table S2 Environmental variables projected to predict the potential global suitable habitats (PGSH) of *Sorghum halepense.*

Variable	Description	Unit
bio1	Annual mean temperature	℃
bio2	Mean diurnal range (Mean of monthly (max temp-min temp)	℃

bio3	Isothermality (Bio2/Bio7)×100	-
bio4	Temperature seasonality (standard deviation×100)	℃
bio5	Max temperature of warmest month	℃
bio6	Min temperature of coldest month	℃
bio7	Temperature annual range (bio5-bio6)	℃
bio8	Mean temperature of wettest quarter	℃
bio9	Mean temperature of driest quarter	℃
bio10	Mean temperature of warmest quarter	℃
bio11	Mean temperature of coldest quarter	℃
bio12	Annual precipitation	mm
bio13	Precipitation of wettest month	mm
bio14	precipitation of driest month	mm
bio15	Precipitation seasonality (coeffcient of variation)	-
bio16	Precipitation of wettest quarter	mm
bio17	Precipitation of driest quarter	mm
bio18	Precipitation of warmest quarter	mm
bio19	Precipitation of coldest quarter	mm
LUC	Land use change	
